# The Challenge of Developing Entrepreneurial Competence in the University Using the Project-Oriented Learning Methodology

**DOI:** 10.3389/fpsyg.2022.966064

**Published:** 2022-07-22

**Authors:** Paula Crespí, Marián Queiruga-Dios, Araceli Queiruga-Dios

**Affiliations:** ^1^Facultad de Educación, Instituto de Acompañamiento, Universidad Francisco de Vitoria, Madrid, Spain; ^2^Facultad de Comunicación, Instituto de Acompañamiento, Universidad Francisco de Vitoria, Madrid, Spain; ^3^Departamento de Matemática Aplicada, Universidad de Salamanca, Salamanca, Spain

**Keywords:** entrepreneurial competence, self-awareness, teamwork, communication, project-oriented learning, higher education

## Abstract

One of the objectives of the United Nations and the European Commission is to encourage the implementation of education plans and strategies to develop entrepreneurial competence. This refers to the ability to identify needs or discover opportunities and to act on them in order to create value for society. This paper aims to demonstrate the impact of the project-oriented learning (POL) methodology on the development of certain generic or transversal competences associated with entrepreneurship among first-year university students in Madrid. The competences associated with entrepreneurship analyzed in this work are: self-awareness, self-reliance, achievement orientation, proactivity, cooperative teamwork, team management, planning and organization by objectives and communication. The study used a single-group pre-test/post-test, quasi-experimental methodology with a sample of over 300 students of the Francisco de Vitoria University (UFV). The results show a significant increase in self-perceived development of transversal competences associated with entrepreneurship. These findings confirm the main hypothesis that POL is an ideal methodology for the development of transversal competences associated with entrepreneurship.

## Introduction

### Entrepreneurship and Entrepreneurial Competence

The term entrepreneurship is closely associated with the world of business, start-ups and enterprise. Thus, the goals of entrepreneurship have traditionally been associated with creating wealth, managing risks and uncertainty, identifying opportunities and learning from experience (Vaicekauskaite and Valackiene, 2018).

Comisión de Cultura y Educación (2015) considers entrepreneurship and the spirit of enterprise as key strategic competences for lifelong learning.

According to Hahn et al. (2017), there is no general consensus on the exact definition of entrepreneurial competence within the scientific community but a number of studies associate this competence with the ability to contribute to society by identifying and acting on perceived opportunities (Mwasalwiba, 2010; Bacigalupo et al., 2016). Expanding on this, entrepreneurial competence can be understood as the ability to conceive of new ideas, identify opportunities and to act on them in order to create value for society (Bacigalupo et al., 2016; Gianesini et al., 2018; Renfors, 2020). According to Comisión de Cultura y Educación (2015) a fundamental aspect of this competence is the transformation of ideas into real action.

Entrepreneurial competence has recently been defined as a set of skills, attitudes and knowledge for innovation, creativity, leadership and the ability to seize opportunities. This competence promotes problem solving and decision making, positive social attitudes, the ability to explore/exploit opportunities and economic advancement. It is also associated with the ability to apply knowledge and implement ideas in different areas of life, initiative, problem identification and problem solving, the ability to react and adapt to change and reasonable risk-taking. An entrepreneurial person can create value for others in all areas of life (Montes-Martínez and Ramírez-Montoya, 2020).

Entrepreneurial competence is most often categorized as generic or transversal. According to Tobón (2013), transversal competences are “fundamental to achieve personal fulfillment, manage projects, contribute to ecological balance and act in any occupation, job and/or profession” (p. 113). Although not all authors agree on the key competences within the entrepreneurship meta-competency, these have been broadly identified as: identification and evaluation of opportunities, innovation and creativity, planning-organization, proactivity, achievement orientation, locus of control, self-awareness, communication (listening), cooperative work, adversity management and team-people management (Neary et al., 2015; Bacigalupo et al., 2016; Comisión Europea, 2016).

Thus, this competence is not limited merely to the business world but has much broader and transversal aspects (Núñez and Núñez, 2016) that contribute to personal growth and social progress, enhance employability and foster innovation and enterprise (Bacigalupo et al., 2016).

### Entrepreneurship Education

Entrepreneurship Education (EE) has become a policy priority, especially in vocational and higher education (Komarkova et al., 2015). The European Commission considers entrepreneurship as a set of key competences for employment, social inclusion, active citizenship and personal development that can be instilled at all educational levels (Bacigalupo et al., 2016).

Thus, EE is conceived as a fundamental concept to further the development of entrepreneurial skills, attitudes and competences at all educational levels and in ways which are beneficial to society.

In higher education, EE specifically relates to professional knowledge and skills that will allow graduates to participate and make a valuable contribution to society (Lilleväli and Täks, 2017; Lindner, 2018).

Depending on the specific objectives, EE may be delivered in a number of ways. There is no universal pedagogical methodology in EE and the choice of learning techniques and tools depends on the target audience and the limitations or restrictions imposed by institutions. However, active methods are considered the most effective, including case studies, gamification, mentoring, networking, problem-solving methodologies, design thinking, business analysis, entrepreneurship camps, etc. (San Tan and Ng, 2006; Bager, 2011; Nielsen and Stovang, 2015; Lackéus, 2020).

Most researchers agree that Project-Oriented Learning (POL) is a highly effective teaching method for EE, including business simulations, role-playing, industrial/professional training, etc. (Arasti et al., 2012).

### Project-Oriented Learning and Its Impact on the Development of Entrepreneurial Competences

Project-oriented learning (POL) stems from the notion of education as a dynamic process in which the student actively participates in their own learning. Studies have found that learning is the result of students’ actions and that the role of teachers is to facilitate and encourage constructive learning activities. From this perspective, the transmission of content through lectures is highly static, relegating the student to a passive role. For more effective learning, teachers should focus on helping students acquire self-directed learning skills (Gijselaers, 1996; Ruiz-Rosa et al., 2021).

One advantage of POL is that it enables students to learn outside the classroom, associating content and context, theory and practice, and creating opportunities for students to apply their knowledge in a real world context. Through the POL methodology, new knowledge is constructed and learning becomes a constructive process rather than merely receptive (Chinowsky et al., 2006).

POL is a cross-curricular, long-term activity that takes place, in part, outside the classroom; it is an individual, cooperative, student-centered activity. To develop a project, students must collect and analyze data and write about a real-world topic (Carter and Thomas, 1986; Beckett, 2002; Kim, 2015). The POL constitutes a methodology that works on the basis of the acquisition of technical (hard) and transversal (soft) competences; both crucial in the future professional career of the university student. In POL, students work in groups and “learn by doing” to ensure the success of the project designed by the teacher/instructor (Amiel et al., 2014; Iriondo et al., 2019).

Among the active learning methodologies, the POL is among those with the greatest impact on the acquisition of entrepreneurship competences, contributing to the development of instrumental, systemic and interpersonal competences among university students (Ruiz-Rosa et al., 2021).

It has been shown that the use of POL in higher education improves students’ skills and competences such as teamwork, self-confidence, critical thinking, motivation, leadership, etc. (Leite, 2017; Savant and Pawar, 2017). These are all competences which are very necessary in entrepreneurship (Athayde, 2009).

### Research Objectives and Hypotheses

The purpose of this research is to demonstrate the impact of the POL methodology on the development of certain generic competences associated with entrepreneurship among first-year university students. Thus, the following hypotheses are put forward:

H1: There are significant differences in self-perceived development of transversal competences associated with entrepreneurship among first-year students after working with the POL methodology.

H2: There are significant differences in self-perceived development of intrapersonal competences associated with entrepreneurship (“self-awareness,” “self-reliance,” “achievement orientation,” and “proactivity”) among first-year students after working with the POL methodology.

H3: There are significant differences in self-perceived development of interpersonal competences associated with entrepreneurship (“cooperative teamwork,” “team management,” “planning and organization,” and “communication”) among first-year students after working with the POL methodology.

## Methodology

### Research Design and Study Variables

This research project used a single-group, pre-test/post-test, quasi-experimental methodology. The target population was all first-year university students in Madrid (Spain) and was conducted at the Universidad Francisco de Vitoria (UFV) where the impact of the POL methodology on the self-perceived development of transversal competences associated with entrepreneurship was analyzed. An incidental and random sample was used, and the sample size was calculated using the Ene 3.0 statistical program, with a 95% confidence interval, a Standard Deviation (SD) of 3 and a precision level of 0.40. All first-year students enrolled at the UFV (1,315 students) was the starting point. The sample size was subsequently calculated by strata, each stratum being a specific faculty. The Ene 3.0 statistical program indicated an ideal sample size of 227 students. The final sample, after discarding a number of invalid candidates, was a total of 309 first-year students. This sample size is considered adequate and representative of the population under study; it is also representative of the different faculties of the university given that, in all cases, the sample size is equal to or greater than the minimum indicated by the statistical program (Table 1). The sample is also largely homogeneous, consisting entirely of first-year university students, 18 years of age, with a medium to medium-high socio-economic level.

**TABLE 1:** Research sample size.

Faculty	Sample questionnaire	Minimum sample
Experimental sciences	44	25
Education and humanities	48	18
Health sciences	88	77
Legal and economic sciences	50	50
Communication sciences	46	45
Polytechnical school	33	12
Total	309	227

*The authors.*

Project-oriented learning is the most likely cause of the increased self-perceived development of transversal competences associated with entrepreneurship and thus constitutes our primary independent variable. Faculty and gender are the secondary and independent control variables. Finally, the intra- and interpersonal competences associated with entrepreneurship: “self-awareness,” “self-reliance,” “achievement orientation,” “proactivity,” “cooperative teamwork,” “team management,” “team planning-organization,” and “communication,” constitute the dependent variables of our study.

### Project-Oriented Learning Methodology in the Context of a Transversal Subject

The POL is considered the most appropriate methodology for the development of transversal competences associated with entrepreneurship. Students undertake a project in teams that responds to a real need or problem detected in a given time. They must propose a solution or response to that need, deciding on the appropriate actions, creating a plan (the tasks that need to be done and in what order) and organization (assigning tasks to each team member). The project offers students the opportunity to apply the knowledge, skills and attitudes, that is, the competences developed in class through Experiential Learning. Therefore, POL is ideal for putting into practice their transversal competences. It should be noted that this methodology was applied in a course called Personal Skills, which aims to encourage self-discovery and for students to consider themselves as unique, with a valuable contribution to make to society and to foster personal and professional growth. This course is particularly focused on the development of transversal competences.

**FIGURE 1:**
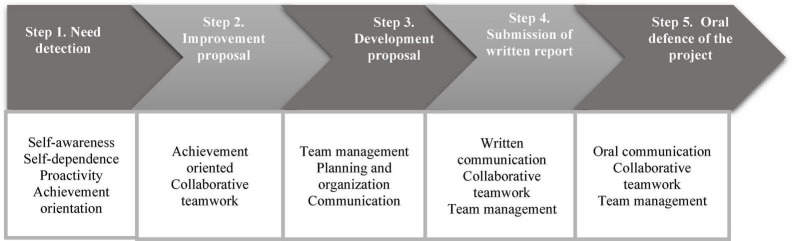
POL phases associated with transversal entrepreneurship competences.

Within this context, POL provides students the opportunity to experience real teamwork with two main objectives: (1) to propose and carry out a real improvement of reality; that is, an improvement that transforms reality into something better. (2) to work both individually and collectively as a team, applying transversal competences. The exercise consists of the following steps (Figure 1):

Step 1. Needs detection. In teams, students first ask themselves about society, its needs, shortcomings and problems. Through a brainstorming exercise, each team member proposes a specific need. At the end of the exercise, each member chooses a topic to work on individually and propose to the team. This implies that each team member investigates a need or problem in depth, who it affects, how it affects them, what their real needs are, etc. Students also devise one or more solutions for that need. In this step students develop the competences of self-awareness, self-reliance, proactivity and achievement orientation. These competences are eminently intrapersonal, precisely because the activity in this first step is eminently individual even though the exercise is ultimately team-based.

Step 2. Improvement proposal. A few weeks later, each team meets and members explain the need they have detected and how they propose to solve it. Once everyone has made their proposal, the team collectively decides and reaches a consensus on what need they want to work on and how they will do it. This is the actual design of the improvement proposal. Team decisions must be by consensus, thus ensuring that everyone has the opportunity to influence the final decision; this implies more than unanimous decision-making where, although everyone agrees, not everyone has the opportunity to contribute. This is a very important factor. It ensures that the POL and associated transversal competences are develop properly. In this step, although the competences of the first step are still present, more work is done on the competences of teamwork and achievement orientation.

Step 3. Development proposal. Once the team has decided by consensus what need it wants to address and how to provide a real solution, it is time to execute the proposal. In other words, students must work to make their proposal a reality that actually helps real people and impacts real lives. This stage generally lasts approximately 4 weeks, developing the competences of team management, planning-organization and communication.

Step 4. Submission of the written report. Once the proposal has been implemented and the results collected, the team must draft a written report of the project. At this stage, each team has to reflect and “narrate” very clearly the whole POL process, considering the main objectives: development of transversal competences associated with entrepreneurship in the context of a project for the transformation/improvement of society. Students are provided with a guide to prepare their report, with an approximate length of 4,500 to 6,000 words. In this step students develop their written communication skills, collaborative teamwork and team management competences.

Step 5. Oral defense of the project. Once the written report is complete, the team prepares its oral defense. They will give a 7 min presentation explaining the project, the objectives and how these were achieved. Students will learn how to create a well-structure, visual and dynamic presentation; and practice their communication skills: tone, rhythm, speed, gestures, look, use of silences, movement in the room, etc. Since it is a team presentation, it will require the entire team to work as one. The following aspects are observed and valued: proper presentation of the team, a coherent discourse among members, team spirit and mutual support throughout the presentation, etc. This step focusses especially on oral communication competences, collaborative teamwork and team management.

Finally, for the POL to be effective, the following aspects should be taken into account: (1) Work teams should be formed on the first day. The classroom should be arranged to facilitate cooperative work; desks should be arranged so that each team can work closely together and see each other’s faces. (2) Teaching days should be alternated, one day using experiential learning to develop key transversal competences, and the other implementing these competences through POL. (3) Create an atmosphere of safety and trust in the classroom and within teams. In this type of active methodologies, where the student is the protagonist of their learning, a climate of trust is essential in which no judgments are made, no labels applied, mistakes allowed, etc. This will encourage students to be authentic and thus favor their learning. (4) Teachers should act as a facilitator of learning, being authentic and open. They will be the reference for the class and therefore must demonstrate the aspects and competences they want to teach. (5) Students must produce a written report, a synthesis of the entire project, reflecting how they responded to the objectives of the project and the results achieved. They will also present an oral defense of the project before a panel of experts in transversal and technical competences, and also before experts in entrepreneurship. It is important for students to give individual and team presentations to facilitate adequate follow-up of each team. (6) The evaluation must be continuous, by assignments and based on rubrics that reflect individual and team development. Evidently, the written report and the oral defense will have the highest weight in grading the course (50%).

### Data Collection

In conducting the study, all participating teachers of the Personal Skills courses of the various faculties and degrees were contacted and informed of the aims of the study. To avoid possible extraneous variables, the same person delivered the questionnaire to the students to ensure instructions were the same. Students received a brief explanation of the study and the Questionnaire on Transversal Competences (QTC). The questionnaire was conducted online using Google Form. Students received a QR code to access the questionnaire which could be easily completed using a PC, tablet or smart phone. Prior to submitting their answers, participant completed an informed consent form, allowing their data to be used generically, never individually, ensuring the confidentiality and anonymity of the responses. The QTC was reviewed by UFV Legal Counsel, guaranteeing compliance with current data protection regulations (Reglamento 2016/679, 2016; Reglamento 2018/1725, 2018).

### Data Analysis

For descriptive analyses, the mean, variance, standard deviation, and range were calculated. For the correlation analysis, Pearson’s correlation coefficient was calculated. For the hypothesis contrast, the Student’s *t*-test and effect size (Cohen’s *d*) estimations were calculated.

### Measurement Instrument

The study used the “Questionnaire on Transversal Competences” (QTC), with a Likert-type scale from 1 to 6, with 1 being the lowest and 6 being the highest score (Crespí, 2019). The QTC was developed based on other questionnaires (Pérez et al., 2010; Morales et al., 2013; Pozo, 2017; Ruiz et al., 2017). The QTC was validated by a panel of experts, who modified the wording of some items. The final version of the questionnaire (Supplementary Annex 1) consisted of 16 items. The first 4 items collect student information about the university, gender, age, and studies. The following 12 items refer to behaviors associated with each competency. The first 6 refer to behaviors associated with intrapersonal transversal competences and the following 6 with interpersonal ones. Being a 12-item questionnaire, with a rating scale from 1 to 6, the total score of the questionnaire can range from a minimum of 12 points to a maximum of 72 points. The questionnaire has four major sub-dimensions: knowledge, self-improvement, teamwork, and communication.

The reliability analyses (Table 2) reflect excellent overall internal consistency (0.90) at pre-test time and a good a test-retest correlation (0.77). The linear correlations are considered significant and relevant. In all cases, the validity and item homogeneity analyses showed indices >0.20, considered satisfactory. Exploratory factor analysis (EFA) was also carried out using factorization and rotation methods: ACP and Oblimin. On the whole, EFA corroborated the dimensional structure of the questionnaire (Crespí, 2019).

**TABLE 2:** Reliability (internal consistency and test-retest correlation) analysis of the QTC.

	Global	Intrapersonal dimension	Interpersonal dimension	Knowledge subdimension	Self-improvement subdimension	Teamwork subdimension	Communication subdimension
No. of items	12	6	6	3	3	3	3
Cronbach’s α (rxx)	0.90	0.81	0.84	0.70	0.71	0.86	0.75
Test-retest (rxx)	0.77	0.70	0.77	0.72	0.60	0.76	0.78

*Source: Crespí (2019).*

The reliability analyses (Table 2) reflect excellent overall internal consistency (0.90) at pre-test time and a good a test-retest correlation (0.77). The linear correlations are considered significant and relevant. In all cases, the validity and item homogeneity analyses showed indices greater than 0.20, considered satisfactory. An Exploratory factor analysis (EFA) was also carried out using factorization and rotation methods: ACP and Oblimin. On the whole, EFA corroborated the dimensional structure of the questionnaire (Crespí, 2019).

## Results

### Descriptive Analysis

The descriptive analyses, comparing pre-test and post-test results, show differences in the means for the dependent variables in this study, all of them favorable.

### Inferential Analysis

The following are the results of the main hypotheses of the study:

H1: There are significant differences in self-perceived development of transversal competences associated with entrepreneurship among first-year UFV students after working with the POL methodology (Table 3).

**TABLE 3:** Self-perceived development of transversal competences associated with entrepreneurship.

Competences	Initial average	Final average	SD	Mean difference	Student’s t	Sig (bilateral)	Effect size (d)
Total score for transversal competences	40.38	59.59	4.77	19.21	77.20	0.00	0.98

*The authors.*

Table 3 shows the mean difference (beginning and end of the course) among UFV students in their self-perceived development of transversal competences, with an increase in post-test results of 19.21. The t-statistic is greater than 1 with a bilateral critical level of less than 0.05 and can therefore be considered significant. This corroborates our hypothesis about the existence of significant differences in self-perceived development of transversal competences associated with entrepreneurship among first-year UFV students after working with the POL methodology. The effect size is large (0.98), following the assessment guidelines of Pardo and San Martín (2015), confirming that these differences are due to the impact of the POL methodology.

H2: There are significant differences in self-perceived development of intrapersonal transversal competences associated with entrepreneurship (“self-awareness,” “self-reliance,” “achievement orientation” and “proactivity”) among first-year students after working with the POL methodology.

Table 4 shows the mean difference among UFV students in their self-perceived development of intrapersonal competences, with an increase of 10.43 points. The t-statistic (66.66) and bilateral significance (0.00) indicate significant differences between the beginning of the course and the end. The effect size (0.97) is large (Pardo and San Martín, 2015), and it is therefore assumed that these differences are due to POL.

**TABLE 4:** Self-perceived development of intrapersonal competences associated with entrepreneurship.

Competences	Initial average	Final average	SD	Mean difference	Student’s t	Sig (bilateral)	Effect size (d)
Total score for intrapersonal competences	19.74	30.17	2.75	10.43	66.66	0.00	0.97
Self-awareness	2.91	5.00	0.71	2.09	51.73	0.00	0.95
Self-dependence. Locus of control	3.56	5.06	0.65	1.5	40.44	0.00	0.92
Achievement oriented	3.36	4.97	0.81	1.61	35.03	0.00	0.89
Proactivity	3.39	5.05	0.74	1.66	39.49	0.00	0.91

*The authors.*

Similar results were found for all analyzed intrapersonal competences: “self-awareness,” “self-reliance,” “achievement orientation” and “proactivity,” with significant differences in all competences largely due to POL. With regards to the difference in means, the Student’s *t*-test and effect size, the competency with the greatest difference is “self-awareness,” followed by “self-reliance” and “proactivity.”

H3: There are significant differences in self-perceived development of interpersonal competences associated with entrepreneurship (“cooperative teamwork,” “team management,” “planning and organization,” and “communication”) among first-year students after working with the POL methodology.

In line with the results of the previous hypothesis, Table 5 shows significant differences in the total score for interpersonal competences and the specific competences “cooperative teamwork,” “team management,” “planning and organization,” and “communication.” The effect size (≥ 0.89) again points to POL as the main driver of this change.

**TABLE 5:** Self-perceived development of interpersonal competences associated with entrepreneurship.

Competences	Initial average	Final average	SD	Mean difference	Student’s t	Sig (bilateral)	Effect size (d)
Total score for interpersonal competences	20.63	29.41	2.56	8.78	60.28	0.00	0.96
Cooperative teamwork	3.28	4.96	0.80	1.68	36.89	0.00	0.90
Team management	3.63	5.05	0.68	1.42	36.55	0.00	0.90
Planning and organization	3.54	4.94	0.70	1.40	35.21	0.00	0.89
Communication: empathy, assertiveness and listening	3.68	5.12	0.70	1.44	36.33	0.00	0.90	

*The authors.*

## Discussion

The development of entrepreneurial competences in education must be considered a structural and universal need, that is, one that can be addressed through any branch of knowledge or discipline. The key is to enable “people to be creative and entrepreneurial” (Tobón, 2013, p. 152). According to Comisión Europea (2013) and the Global Entrepreneurship Monitor Spain (GEM, 2020), it is essential to develop training programs, seminars or curricular subjects that encourage and foster the development of entrepreneurial competences (Nabi et al., 2017).

A number of studies into the relationship between education and entrepreneurship suggest that those who start their own businesses generally have a higher level of education than those who do not (GEM, 2020). However, findings also suggest that formal education itself does not necessarily foster entrepreneurship but rather prepares students to function within a corporate environment that actually suppresses creativity and entrepreneurship. Thus, specialized courses that foster entrepreneurship have become increasingly common within higher education, with ever greater emphasis on EE (Peterman and Kennedy, 2003).

Although EE is considered essential to promote sustainable economic growth, there is no consensus on the appropriate educational model. There is a consensus, however, on the need for something other than traditional management courses. Hence, a great deal of research has been done in identifying the pedagogical methodologies which are most effective in encouraging entrepreneurial and enterprising behavior among students. Teaching entrepreneurship requires use, application, action and practice (Neck and Greene, 2011; Kassean et al., 2015).

According to Lackéus (2018), the new approach to EE is fundamentally about enabling students to learn by applying their knowledge in creating something of value for others. Empirical studies have shown that such an approach favors the development of entrepreneurial competences, deep integration into core curricula and alignment with the humanistic values of many teachers.

The current educational paradigm places students as the protagonists of their own learning, that is, as active agents in the education process. POL provides the necessary tools to develop both EE and entrepreneurial competences. In fact, there are cases in which POL has been used to create a real company (Edelman et al., 2008), with real action plans and simulations addressing real-world needs (Kuratko, 2005) or solving real-world problems (San Tan and Ng, 2006). A study by Pepin and St-Jean (2018) found significant improvements in both transversal and interpersonal competences as a result of an entrepreneurship project developing entrepreneurial competences such as creativity, achievement and self-control and most notably in leadership.

Thus, entrepreneurship competences can make a significant contribution to student learning when embedded within generic or transversal competences and developed as an essential competence in education (Cárdenas and Montoro, 2017; Paños-Castro and Arruti, 2019). In this sense, as noted above, the Comisión Europea (2013) has emphasized the need to incorporate specific subjects or training programs aimed directly at the development of entrepreneurial skills.

In addressing this need, this study analyzes the impact of the POL methodology on the development of specific competences associated with the meta-competency of entrepreneurship. The results from our main hypotheses confirm the findings of previous research, specifically regarding the effectiveness of POL as a methodology for the development of transversal or generic competences (Wee, 2004; Ruiz-Rosa et al., 2021). Our first hypothesis (Table 3) confirms the existence of significant differences in self-perceived development of transversal competences associated with entrepreneurship among first-year UFV students after working with the POL methodology in a traversal subject (very large effect size). Significant differences are observed when comparing the initial, pre-test moment, prior to working with POL, and a final, post-test moment after implementation. The second hypothesis (Table 4), in line with the results of the first hypothesis, confirms the positive impact of POL on the development of “intrapersonal competences” on the whole, as well as “self-awareness,” “self-reliance,” “achievement orientation” and “proactivity” (significant differences in all cases and large effect size). The third hypothesis (Table 5) again confirms the impact of the POL methodology on the development of competences, in this case, “interpersonal competences” on the whole, as well as “cooperative teamwork,” “team management,” “planning and organization,” and “communication: empathy, assertiveness and listening.” Therefore, it can be concluded that these improvements in self-perceived development of these competence is largely due to POL.

As noted above, the Personal Skills course, using the POL methodology, attempts to address the call by the European Commission and the European Parliament Committee on Culture and Education, among others (Comisión Europea, 2013, 2016; Comisión de Cultura y Educación, 2015; Bacigalupo et al., 2016). In offers an innovative solution, at least partially, to current calls for EE and the need to develop entrepreneurial competences for the personal and professional future of students. The course has had a significant impact on the development of entrepreneurial competences such as “self-awareness,” “self-reliance,” and “proactivity” followed by “collaborative teamwork,” “team management,” “communication,” “planning and organization,” and “achievement orientation.”

This study, for now, is difficult to replicate as it is designed since few universities have a specific subject whose content is dedicated exclusively to the explanation, development, and implementation of transversal competences. However, it would be of great interest to carry out a comparative study (independently of the subject) on the effectiveness of the POL in the development of entrepreneurial competence in favor of EE training; especially in teamwork whose teachers’ involvement would include sufficient explanations of what this competence implies. It is noted, in any case, that the university community has yet to fully explore new ways to further the development of entrepreneurial, transversal skills that are essential for the personal and professional future of their students.

## Data Availability Statement

The raw data supporting the conclusions of this article will be made available by the authors, without undue reservation.

## Ethics Statement

Ethical review and approval was not required for the study on human participants in accordance with the local legislation and institutional requirements. The patients/participants provided their written informed consent to participate in this study.

## Author Contributions

MQ-D has mainly coordinated the research tasks related to the documentation of the POL learning methodology system, its relationship and impact on the development of competencies in the university environment as well as specifically on the entrepreneurship competency, and carried out a small systematic review of all recent articles on this topic and has contributed to discuss the results of the study. PC and AQ-D have mainly coordinated the research tasks related to the quantitative part of the study, sample selection, statistical analysis, and research results. All authors contributed to the article and approved the submitted version.

## Conflict of Interest

The authors declare that the research was conducted in the absence of any commercial or financial relationships that could be construed as a potential conflict of interest.

## Publisher’s Note

All claims expressed in this article are solely those of the authors and do not necessarily represent those of their affiliated organizations, or those of the publisher, the editors and the reviewers. Any product that may be evaluated in this article, or claim that may be made by its manufacturer, is not guaranteed or endorsed by the publisher.
