# Supplementary material for: The Challenge of Developing Entrepreneurial Competence in the University Using the Project-Oriented Learning Methodology
Source: Front Psychol. 2022 Jul 22;13:966064. doi: 10.3389/fpsyg.2022.966064 (PMC9355501; doi:10.3389/fpsyg.2022.966064)
Supplement: Supplementary file 1 [file Table_1.pdf]

Annex 1. Questionnaire on personal competences

This questionnaire is anonymous. Its objective is to know your level of development in some important personal competencies in university education: knowledge and personal development, teamwork and communication. Please mark the option with which you best identify yourself for each item. The rating scale is as follows: 1 Not at all 2 A little 3 Regular 4 Quite a lot 5 A lot 6 Completely

Thank you for your time and truthfulness in your answers. It is important that you answer the questions based on how you are in general, not on how you feel today, or how you would like to be or feel. If you do not understand a question, please discuss it with the teacher in charge. The answers are guaranteed to be completely confidential.

1. University:
2. Sex: □ Female (1) □ Male (2)
3. Age:
4. Grade:
5. Ability to know how and who I am (strengths, weaknesses, gift, vocation, mission).
6. Ability to accept and love myself as I am.
7. Ability to respond to the consequences of my actions or decisions.
8. Ability to ask myself or reflect on the big questions of life.
9. Ability to set goals that allow me to develop and improve myself.
10. Ability to take actions that allow me to achieve my goals, dreams, etc.
11. Ability to work and encourage all members of the team to contribute.
12. Ability to encourage and work in a team with respect, positive attitude, motivation.
13. Ability to work and encourage team planning, role sharing and task accomplishment to achieve established objectives.
14. Ability to use language as a means of expression, oral or written.
15. Ability to use para-verbal and non-verbal codes that facilitate communication (tone, rhythm, eye contact, body movement, gestures).
16. Ability to implement: empathy, assertiveness and active listening.
